# Examination of Various Abutment Designs Behavior Depending on Load Using Finite Element Analysis

**DOI:** 10.3390/biomimetics9080498

**Published:** 2024-08-16

**Authors:** Mehmet Onur Yağır, Şaduman Şen, Uğur Şen

**Affiliations:** 1 Electronics and Automation Program, Adapazarı Vocational School, Sakarya University, 54050 Sakarya, Turkey; 2 Dental Implant Design and Application Lab, Sakarya University, 54050 Sakarya, Turkey; 3 Metallurgical Materials Engineering, Faculty of Engineering, Sakarya University, 54050 Sakarya, Turkey; sdmnsen@sakarya.edu.tr (Ş.Ş.); ugursen@sakarya.edu.tr (U.Ş.)

**Keywords:** PEEK abutment, polyetheretherketone, alternative abutment design, abutment force analysis, screw abutment

## Abstract

Studies on dental implant abutments’ geometric design and material selection offer significant innovations and results. These studies aim to improve the abutments’ functionality and aesthetic performance, minimize microcavities’ formation, and ensure implant-supported prostheses’ longevity. For example, CAD-CAM fabricated custom abutments have been found to produce a better marginal fit and fewer microgaps than standard abutments. In an in vitro study, transepithelial abutments offered lower microgap values than titanium-based abutments and provided a better fit at the implant–abutment interface. It is known that studies to improve mechanical and biological performance with Polyether Ether Ketone (PEEK) material have been addressed. New materials such as PEEK and zirconia have offered significant advantages in biocompatibility and aesthetics. Along with those studies, different abutment designs are also important. Abutment geometry is optimized to improve stress distribution and minimize peri-implant bone loss. In implant and abutment connections with different angles, mechanical life performances may vary depending on static and dynamic load. These studies emphasize the importance of material research on different types of connections to improve dental implants’ durability, homogeneous load distribution, and reliability. The abutment parts used in implant treatment are insufficient to distribute the load homogeneously against chewing pressure due to their materials and geometry. Non-uniform load distribution damages the abutment and the prosthetic crown, accelerating the wear process. This study aimed to create different abutment designs to improve dental implants’ biomechanical performance and longevity. This study aimed to increase the mechanical durability of the implant–abutment connection by reducing stress concentrations in response to masticatory compression on the abutment in different directions and forces and to guarantee the long-term success of the implant system by providing a more homogeneous stress distribution. It aimed to apply different forces in the axial direction to these models in a simulation environment and to calculate and compare the deformation and stress load distribution. As a method, three-dimensional models of the parts used in implant treatments and forming the implant system were designed. Different abutment designs were created with these models. Taking the current material values used in implant treatments as a reference, finite element analysis (FEA) was performed by applying different axial loads to each implant system model in the ANSYS software (version 24.1). Comparative analysis graphs were prepared and interpreted for the stress values obtained after the applied load. This study evaluated the mechanical performance of different abutment models (A, B, C, D, and E) under a 100 N load using the Kruskal–Wallis test. The Kruskal–Wallis test showed significant differences between the groups (*p* < 0.001). The greatest difference was observed between models E and A (q′ = 6.215), with a significant difference also found between models C and A (q′ = 3.219, *p* < 0.005). Regarding stress values, the highest stress on the abutment was observed in Model B (97.4 MPa), while the lowest stress was observed in Model E (9.6 MPa). The crown exhibited the highest stress in Model B (22.7 MPa) and the lowest in Model E (17.3 MPa). The implant stress was highest in Model C (14.8 MPa) and lowest in Model B (11.3 MPa). The stress values for the cortical bone and cancellous bone were quite similar across the models, showing no significant differences. These findings indicate that the abutment design and material selection significantly impact mechanical performance. Among the implant systems created with five different abutment models, in which the existing abutment geometry was also compared, homogeneous and axial distribution of the load on the abutment was achieved, especially with viscoelastic and surface area increased abutment designs. Clinically, the inadequacy and limited mounting surface or geometry of the abutments used in today’s implant treatment applications have led to different design searches. It was concluded that the designs in this study, which are considered alternatives to existing abutment models, contribute positively to the mechanical life of the abutment material, considering the von Mises stresses and directions. This study brings a new perspective to today’s practices and offers an alternative to treatment practices.

## 1. Introduction

In recent years, various abutment designs and materials have been studied to enhance the success of dental implants. Abutments manufactured with CAD-CAM technology offer better marginal fit and reduced microgaps compared to standard abutments [1,2]. New materials such as PEEK and zirconia provide significant advantages in biocompatibility and aesthetics [3,4]. Additionally, optimizing abutment geometry helps improve stress distribution and reduce peri-implant bone loss [5,6].

Dental implants are a standard medical solution to treat extracted or lost teeth [7,8]. Dental implant treatment is a process that requires the integration of various parts. Among these parts, the first is the titanium implant, which is surgically placed in the jawbone [9,10]. The abutment is the connection part used in dental implant treatment and is fixed on the implant with a screw [7,11]. The abutment attached to the implant placed in the jawbone connects with the dental prosthesis. This way, the dental prosthesis is firmly attached to the implant and is expected to function like natural teeth. The abutment contributes to the longevity of the implant by distributing the force applied to the jawbone. Usually made of metal, ceramic, or porcelain, abutments can be in various shapes and sizes. Abutment selection is chosen according to the patient’s needs and the type of implant [12,13]. Titanium alloys are the most frequently preferred material type as abutment material due to their excellent biocompatibility [14,15,16]. This is due to titanium abutments’ durability and long life. As a result, choosing the right abutment is essential for the crown installation and, therefore, the successful completion of the implant treatment.

Many researchers have created literature on dental implants and restorative dentistry and work on abutment design. Dr. Carlo E. Poggio, MD, Frank Schwarz, MD, Avinash S. Bidra, and doctor researchers such as Paulo G. Coelho are some of the prominent names in this regard [17,18,19]. When the studies in the literature are evaluated, it is revealed that the abutment design’s development, durability, and functionality are at least as important as the implant.

In this study, different abutment geometries were designed based on literature studies to reduce the stress concentration on the implant and, at the same time, provide an alternative to abutment and crown installation. Homogeneous and axial distribution of the load has been achieved, especially with the abutment design with increased surface area by the viscoelastic material. For viscoelastic abutment materials, H. Chang et al. confirmed that the periodontium of natural teeth has viscoelastic properties such as creep, stress relaxation, and hysteresis, thus providing more energy consumption than dental implants [20]. The study revealed the importance of viscoelastic removable abutment parts for natural tooth movement. However, it can also be inferred that natural dental implant bridges can be made successfully with viscoelastic behavior.

## 2. Materials and Methods

**Figure 1:**
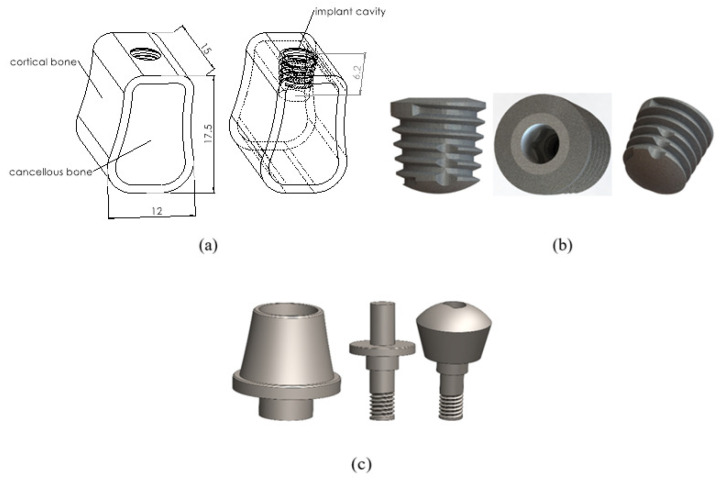
(**a**) Bone cross-section model designed by the dental anatomy and periodontium parts of the jawbone; (**b**) Solidworks design image of the “copaSKY implant 4.0 longueur 5.2 mm” implant; (**c**) Three-dimensional abutment models used in the analysis.

Firstly, a literature search was conducted to determine the material mechanical properties of the parts of the implant systems to be used in finite element analysis. An average value was determined for the implant, abutment, and crown parts that make up the implant system, taking these data from the literature as a reference. In the second stage, three-dimensional models of all parts of the implant system used in the analysis were designed with the SOLIDWORKS 2021 program (Figure 1). After the modeling phase, the models were transferred to the ANSYS program for finite element analysis. Analysis was conducted in the ANSYS environment after material and part contact connections were made with different force values. The following topics provide information about the features and material selection of the parts of the models used in the analysis.

### 2.1. Bone Model and Material

The bone model is a three-dimensional solid model used to assemble implant systems. The bone model was modeled in two layers, cortical and cancellous bone, by the periodontium parts of the jawbone. Since the same force and analysis conditions apply to different implant systems in this analysis study, the bone model was designed to be simplified (Figure 1a). This model is 12 mm wide, 17.5 mm high, and 15 mm deep. The model also has a Ø6.2 mm deep slot for implant mounting. For the ANSYS material definition of the bone model, average elasticity (E) and Poisson (ν) values were determined by taking the cortical bone structure in the literature as a reference (Table 1).

### 2.2. Implant Model and Material

Ti6Al4V alloy is mainly used in dental implants for biocompatibility, strength, and durability. In this study, the implant material was chosen as Ti6Al4V. Copa SKY implant was obtained from Bredent Medical Company for the three-dimensional modeling of the implant to be used in this analysis. The reference code of the implant is Copa 4005. The production code is given as copaSKY implant 4.0 longueur 5.2 mm, and the material content is known as titan grade 4 KV osseo-connect-surface (OCS). The design of the implant model is shown in Figure 1b. For the ANSYS material definition of the implant model, average elasticity (E) and Poisson (ν) values were determined by referring to the literature (Table 1).

### 2.3. Abutment Model and Material

As shown in Figure 1c, different abutment models were created with a distinct and innovative approach based on homogeneous force distribution. In the literature, abutment materials generally include various materials such as titanium, steel, porcelain, ceramic, and zirconium. Titanium is preferred due to its light weight, high strength, corrosion resistance, and biocompatibility. This study chose the abutment material Ti6Al4V in models A, B, C, and D and PEEK, a viscoelastic material, in model E. Viscoelastic materials are known for their energy dissipation (damping) properties and are used to control vibrations and withstand impact loading [29,30]. They can also change shape over time under an applied load and gradually return to their original shape when the load is removed. PEEK is used in oral implantology and prosthodontics to improve the bioactivity and biocompatibility of dental implants [31]. This biocompatible material will provide viscoelastic behavior under load, specific to the designed geometry [32,33,34]. For the ANSYS material definition of the abutment model, average elasticity (E) and Poisson (ν) values were determined by referring to the literature (Table 1).

### 2.4. Three-Dimensional Models of Implant Systems with Different Abutment Designs

**Figure 2:**
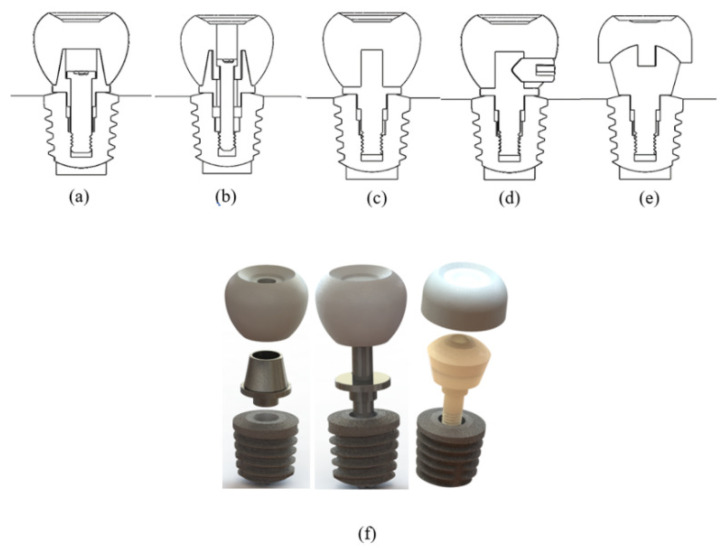
Brief descriptions of the assembly systems shown in the cross-section are as follows: (**a**) In the A implant system, the abutment is screwed into the implant, and the crown is glued to the abutment. (**b**) In the B implant system, both the abutment and crown are screwed into the implant. (**c**) In the C implant system, the abutment is screwed into the implant, and the crown is glued to the abutment. (**d**) In the D implant system, the abutment is screwed into the implant, and the crown is secured to the abutment with a set screw. (**e**) In the E implant system, the abutment is screwed into the implant, and the crown is glued to the abutment. (**f**) The mounting directions of the various abutment designs shown in Figure 1c are illustrated in three dimensions.

**Figure 3:**
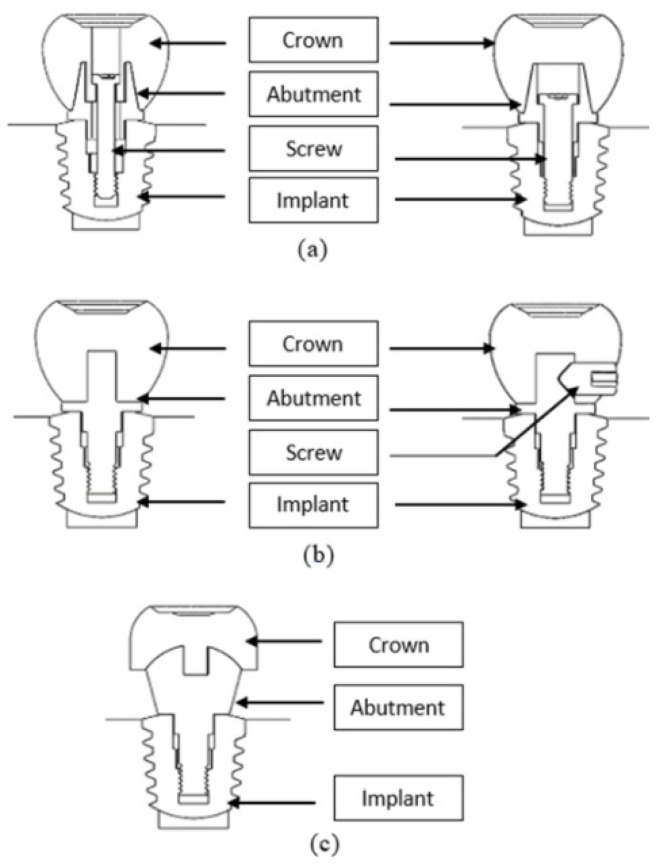
The parts of (**a**) A and B abutment mounting systems, (**b**) C and D abutment mounting systems, (**c**) E abutment mounting system.

Differently designed abutment models created five implant mounting systems (Figure 2). The mounting system in Figure 2a,b is used in current implant treatments. The difference between Figure 2a,b is that the screwing process is done through the crown part. Figure 2c–e show implant assemblies created with alternative abutment geometries. Figure 2c aims to increase the contact surface of the crown on the abutment. The difference between Figure 2c,d is that the crown is screwed to the abutment from the lateral surface. In Figure 2e, viscoelastic abutment material was used. In this model, unlike other models, a geometry similar to the femoral head was taken as reference, and the aim was to distribute the load homogeneously to prevent jawbone resorption and load imbalance between a rigid structure such as an implant and the tooth in single implant applications. To make an easy comparison in analysis studies, the systems of the designed abutment models were named A, B, C, D, and E systems. They were compared with these names in the analysis graphics. The parts used in systems A and B are shown in Figure 3a, the parts used in systems C and D are shown in Figure 3b, and the parts used in system E are shown in Figure 3e.

### 2.5. Finite Element Analysis

**Figure 4:**
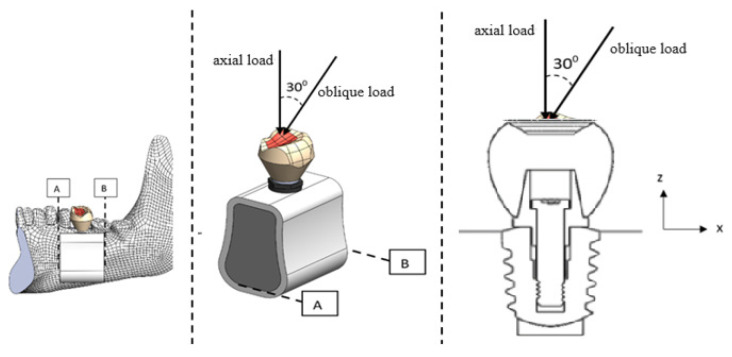
Bone analysis cross-section showing axial and oblique force direction.

Three-dimensional models of the implant systems (Figure 2) designed with the help of the Solidworks 2021 program were saved in Parasolid format. Models in Parasolid format were transferred to the finite element analysis program (ANSYS Workbench 2021 R2, ANSYS Inc., Houston, TX, USA). In finite element (FEA) analysis, the analysis type was selected structural, the solution target was chosen as Mechanical APDL, the number of steps was set as 1 s, and the step end time was set as 5 s. The initial Time Step was determined as 0.5 s, the Minimum Time Step was 0.2 s, and the Maximum Time Step was 0.6 s. In mesh details, the preference for physics was selected as mechanical. Element size was taken as 0.25 mm. A total of 183,053 nodes and 105,715 elements were used to create the finite element model. For each implant system alternative in Figure 3, loads of 100 N, 200 N, 300 N, and 400 N were applied in the axial and oblique directions, as shown in Figure 4. The applied forces, stress distributions on the crown, abutment, implant, and cortical bone, and total displacement amounts were calculated.

### 2.6. Statistical Analysis

Many programs are used for data analysis in the literature studies. One of these programs is SigmaPlot. This program can perform data analysis, statistical tests, or regression analysis [35]. In this study, the Sigmaplot 14.5 program was used for the results obtained by finite element analysis, and a statistical comparison was made for von Mises stress values on abutment models. Another study observed that the relationships and correlations between the analysis values obtained through ANSYS and different variables were obtained in detailed graphics with SigmaPlot [36,37]. Since the number of analyzed data obtained in this study was limited, the Kruskal–Wallis method was selected to determine significant differences between different data sets. The Kruskal–Wallis test is a statistical method that analyzes the median differences between groups and is used in cases that do not meet the assumption of normal distribution. This method was used in a study to analyze the data obtained in ANSYS analysis statistically [38,39]. The Kruskal–Wallis method results in the H statistic and the *p*-value that determines significance.

N is the number of observations in each group, median is the median of each group, and 25% and 75% are the quartiles of each group. It ranks the data and helps determine whether group differences are random. If the *p*-value is less than 0.05, this indicates that there is a statistically significant difference between the groups; if the *p*-value is very small, such as <0.001, the median differences between the groups are very unlikely to be due to chance, and we conclude that these differences are significant. Additionally, in Kruskal–Wallis comparison tables, comparison indicates compared groups, diff of ranks is the difference in ranks between groups, q’ is the critical value, *p* is the *p*-value, which indicates statistical significance, and *p* < 0.050 is if the *p*-value is less than 0.05, which indicates a significant difference.

Sigmaplot 14.5 program Kruskal–Wallis One Way Analysis of Variance on Ranks statistical analysis was prepared for the von Mises (MPa) analysis values obtained at 100 N, 200 N, 300 N, and 400 N load values for the abutment models.

## 3. Results

According to the Kruskal–Wallis test, the H statistic was 49.278, and the *p*-value was <0.001. This indicates that the median differences between the data sets (A, B, C, D, and E) are statistically significant. According to the results of this analysis (Table 2), it was determined that groups E and C showed significant differences in median values from group A. The *p*-value for the E vs. A comparison was <0.001, and the *p*-value for the C vs. A comparison was 0.005, indicating that the median values of these two groups were significantly different from group A. However, the *p*-value for the D vs. A comparison was 0.080, and the *p*-value for the B vs. A comparison was 0.967, indicating that groups D and B were not significantly different from group A. The fact that groups D and B did not show a significant difference suggests that the median values between these groups were similar or not significantly different from the other groups. As a result, it can be concluded that groups E and C are substantially different from the other groups. Still, the median values of the other groups were not significantly different. This suggests that the connection between the surface area and the material between the abutment and crown is essential.

In terms of stress distribution, model E, showing the lowest values depending on the load, had an expected and significant value (*p* < 0.05) due to its viscoelastic behavior (Table 2). In this sense, the surface area was increased at all load values (100 N, 200 N, 300 N, and 400 N), and a statistically significant difference was observed between model E and other abutment models using viscoelastic flexible material.

**Figure 5:**
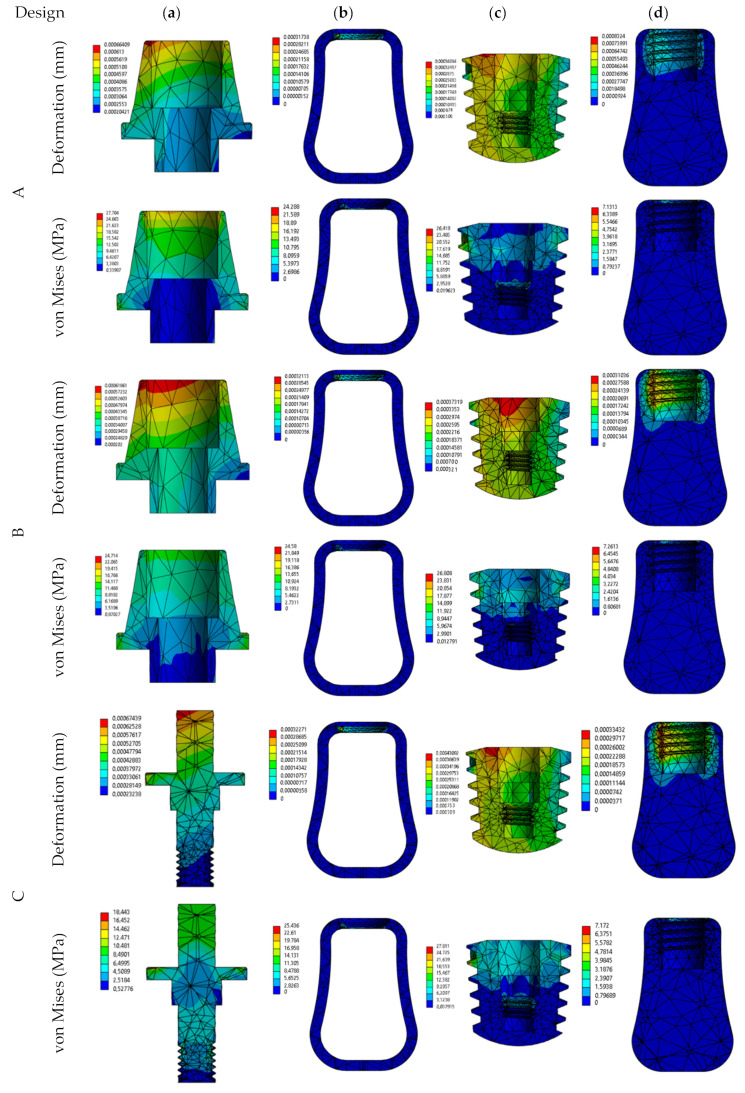

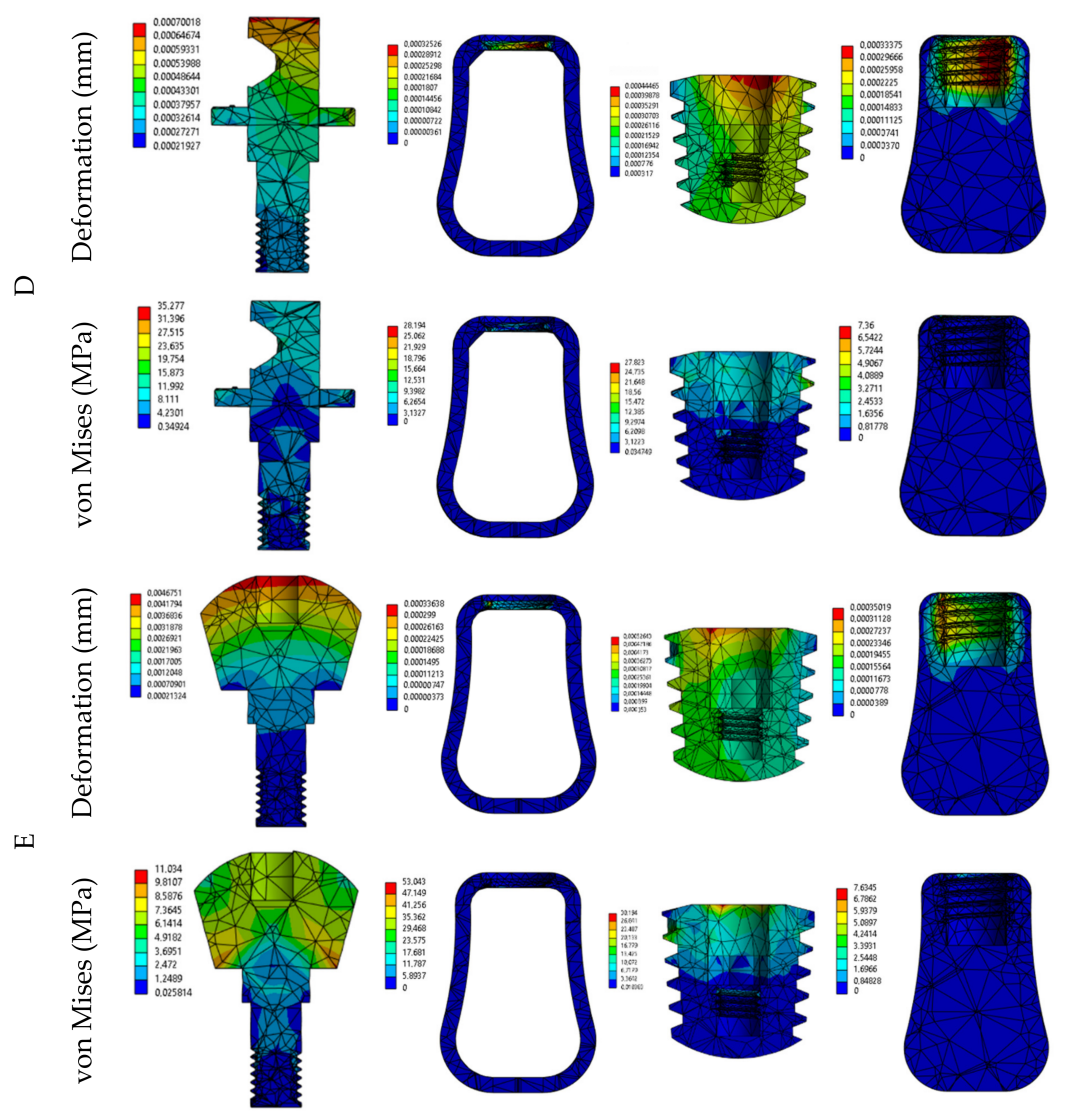
Demonstration of deformation (mm) and von Mises stress (MPa) on the (**a**) abutment, (**b**) cortical bone, (**c**) dental implant, and (**d**) cancellous bone at 100 N for models A, B, C, D, and E.

According to Figure 5a, abutment deformations were seen on the upper surface. Stress tends to be distributed along the upper and lateral surfaces. The stress distribution on the cortical and cancellous bone was seen in the direction of the implant mounting and tooth grooves (Figure 5b,d). The stress distribution on the implant was concentrated near the neck of the cortical bone and also on the tooth grooves (Figure 5c). In addition, when the maximum stress point was taken as a reference, according to Figure 5a, von Mises stress values of 27.7 MPa, 24.7 MPa, 18.4 MPa, 35.2 MPa, and 11.0 MPa were calculated for abutments A, B, C, D, and E, respectively. In this sense, model C, designed as an alternative to the existing abutment (A) with an increased crown mounting surface, and model E, which represents the natural tooth suspension, came to the fore.

The sum of the average deformation values (µm) of each part of the abutment systems during 100 N, 200 N, 300 N, and 400 N axial and oblique loads are shown in Table 3. In Table 3, crown 2.9 µm, abutment 2 µm, implant 1.1 µm, cortical bone 0.1 µm, and cancellous bone 0.4 µm were calculated for model A for the 100 N axial load. In Table 3, crown 14 µm, abutment 6 µm, implant 1 µm, cortical bone 0.2 µm, and cancellous bone 1 µm were calculated for model A for the 100 N oblique load. These deformations increased proportionally at 200 N, 300 N, and 400 N axial and oblique loads.

The sum of the average von Mises values (MPa) in each part of the abutment systems during 100 N, 200 N, 300 N, and 400 N axial and oblique loads are shown in Table 4. In Table 4, crown 21.1 MPa, abutment 11.8 MPa, implant 53.4 MPa, cortical bone 3.2 MPa, and cancellous bone 0.7 MPa were calculated for model A for the 100 N axial load. In Table 4, crown 37.6 MPa, abutment 116.0 MPa, implant 18.3 MPa, cortical bone 6.0 MPa, and cancellous bone 1.1 MPa were calculated for model A for the 100 N oblique load. It was observed that these deformations increased proportionally for the axial and oblique loads of 200 N, 300 N, and 400 N.

In Table 3, crown 2.8 µm, abutment 1.9 µm, implant 1.1 µm, cortical bone 0.1 µm, and cancellous bone 0.4 µm were calculated for model B for the 100 N axial load. In Table 3, crown 13 µm, abutment 5 µm, implant 1 µm, cortical bone 0.1 µm, and cancellous bone 1 µm were calculated for model B for the 100 N oblique load.

In Table 4, crown 22.7 MPa, abutment 47.7 MPa, implant 11.3 MPa, cortical bone 3.2 MPa, and cancellous bone 0.7 MPa were calculated for model B for the 100 N axial load. Table 4 calculates crown 41.8 MPa, abutment 105.9 MPa, implant 17.9 MPa, cortical bone 3.9 MPa, and cancellous bone 1.0 MPa for model B for the 100 N oblique load.

In Table 3, crown 3.3 µm, abutment 1.6 µm, implant 1.2 µm, cortical bone 0.1 µm, and cancellous bone 0.5 µm were calculated for model C for the 100 N axial load. In Table 3, crown 19 µm, abutment 2 µm, implant 2 µm, cortical bone 0.15 µm, and cancellous bone 1 µm were calculated for model C for the 100 N oblique load.

Table 4 calculates crown 19.8 MPa, abutment 24.3 MPa, implant 14.8 MPa, cortical bone 3.2 MPa, and cancellous bone 0.81 MPa for model C for the 100 N axial load. In Table 4, crown 35.1 MPa, abutment 45.3 MPa, implant 23.7 MPa, cortical bone 6.0 MPa, and cancellous bone 1.3 MPa were calculated for model C for the 100 N oblique load.

Table 4 calculates crown 20.4 MPa, abutment 30.7 MPa, implant 12.9 MPa, cortical bone 3.2 MPa, and cancellous bone 0.8 MPa for model D for the 100 N axial load. In Table 4, crown 38.3 MPa, abutment 54.0 MPa, implant 22.4 MPa, cortical bone 6.0 MPa, and cancellous bone 1.3 MPa were calculated for model D for the 100 N oblique load.

In Table 3, crown 24 µm, abutment 4.4 µm, implant 1.2 µm, cortical bone 0.1 µm, and cancellous bone 0.4 µm were calculated for model E for the 100 N axial load. In Table 3, crown 157 µm, abutment 17 µm, implant 2 µm, cortical bone 0.153 µm, and cancellous bone 1 µm were calculated for model E for the 100 N oblique load.

Table 4 calculates crown 17.3 MPa, abutment 9.6 MPa, implant 13.9 MPa, cortical bone 3.2 MPa, and cancellous bone 0.8 MPa for model E for the 100 N axial load. In Table 4, crown 24.6 MPa, abutment 17.0 MPa, implant 23.3 MPa, cortical bone 6.2 MPa, and cancellous bone 1.3 MPa were calculated for model E for the 100 N oblique load.

As a summary of the data in Table 3 and Table 4, model E had generally lower von Mises stress values than the other models. In particular, lower stress values were observed in model E’s crown (17.3 MPa axial, 24.6 MPa oblique) and abutment (9.6 MPa axial, 17.0 MPa oblique) parts compared to the other models. This indicates that the E model causes less stress buildup in these regions.

In the case of the implant part, model E showed moderate stress with 13.9 MPa for the axial load and 23.3 MPa for the oblique load, which places it at a similar or slightly higher level than the other models.

The stress values of the E model for the cortical bone and cancellous bone fragments (3.2 MPa axial, 6.2 MPa oblique, and 0.8 MPa axial, 1.3 MPa oblique) are similar to the other models and do not significantly differ. The E model generally offered lower stress values in the crown and abutment parts, while it showed a balanced performance in the implant and bone parts.

This defines model E as a model with a stress advantage in specific regions and a balanced structure in general. Models C and D had the highest deformation with 2 µm in oblique loads, while models A and B had the lowest values with 1 µm in both load types.

The cortical and cancellous bone deformation values did not differ significantly between the models. Regarding von Mises stress, the highest stress on the crown was observed in model B (41.8 MPa oblique load).

On the abutment, models A and B had the highest stress values (116.0 MPa and 105.9 MPa, respectively). Model A showed the highest stress in axial loads (53.4 MPa) for the implant, while models C and E stood out in oblique loads. Model A had the highest stress on the cortical bone, while the cancellous bone did not significantly differ between the models.

**Figure 6:**
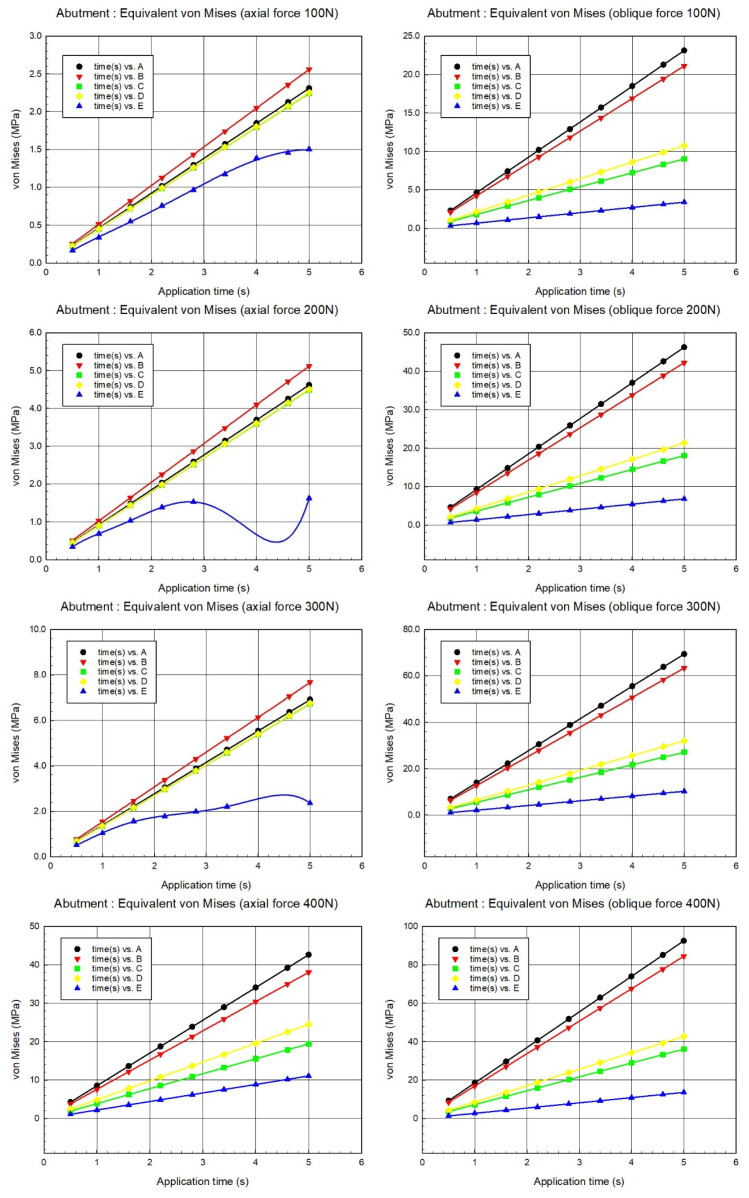
Average von Mises comparison graphs of abutments A, B, C, D, and E at 100 N, 200 N, 300 N, and 400 N axial and oblique load.

In the graphs (Figure 6) where all implant system models are compared at different loads (100 N, 200 N, 300 N, and 400 N), it is seen that the total deformation amounts were generally less in E, and the A, B, C, and D systems follow this model. In addition, it was understood that permanent plastic deformation occurred in the viscoelastic E model due to the increase in force and there was no deformation increase after this point (Figure 6, 400 N).

When Figure 6 is evaluated, the von Mises stress increases as the axial force increases. Model A and model B had the highest stress values at each force level, while model E showed the lowest stress values and fluctuations at some force levels (especially 200 N and 300 N). Model C and model D had lower stress values than A and B but higher than E. This model showed that different abutment designs or material properties can significantly influence stress responses. Under higher forces (300 N and 400 N), the performance of the models becomes more critical; therefore, material and design optimization should be performed to improve durability. The high stress values of models A and B show that these models are more durable, but at the same time, it is necessary to evaluate their fatigue and fracture risks as they can be subjected to higher stresses. According to Figure 6, the von Mises stress increased as the oblique force increased. Case A and case B had the highest stress values at each force level, while case E showed the lowest. Cases C and D had lower stress values than A and B but higher than E.

**Figure 7:**
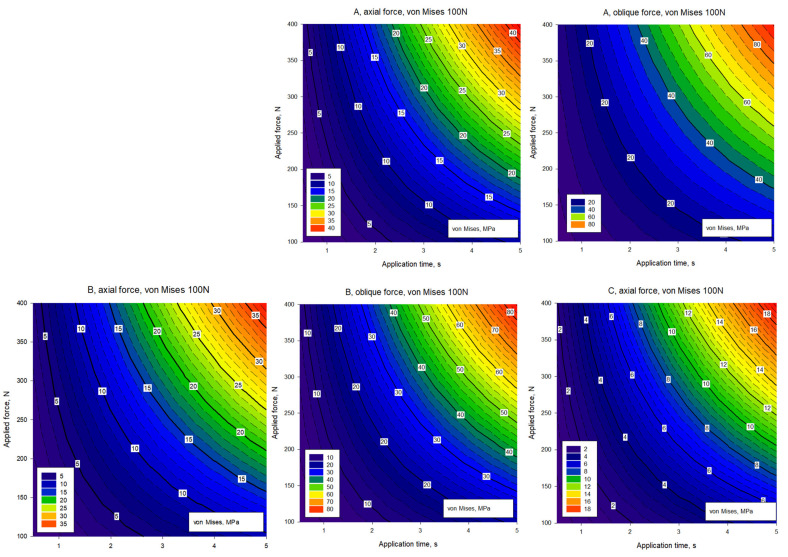

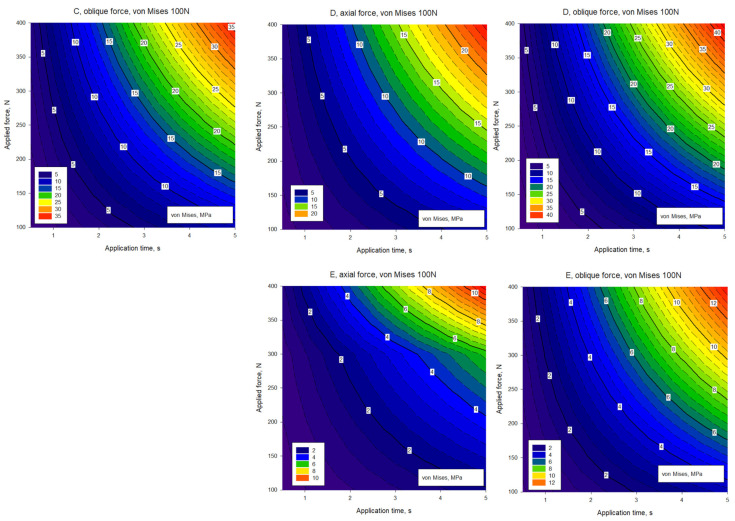
Contour graphs showing the von Mises stress distributions of A, B, C, D, and E implant systems for 100 N, 200 N, 300 N, and 400 N axial and oblique loads.

The abutment models were also evaluated as contour plots. When the von Mises stresses under axial and oblique forces were assessed according to Figure 7, the following observations were made. Model A had the highest von Mises stress under both axial and oblique forces. This was especially evident when a force of 400 N was applied. For axial forces, the stress increased more steadily, while for oblique forces, the stress increased more rapidly. Model B showed slightly lower von Mises stress values compared to model A. However, it was still subjected to high stresses, especially when a force of 400 N was applied. Model C exhibited lower von Mises stresses compared to models A and B. This model can be said to be more durable. While the stress increase was more controlled in axial forces, a more pronounced increase was observed in oblique forces. Model D had one of the lowest values regarding von Mises stress. It showed lower stress than the other models under axial and oblique forces. Model E had the weakest von Mises stress values. It showed less stress than the other models under axial and oblique forces.

**Figure 8:**
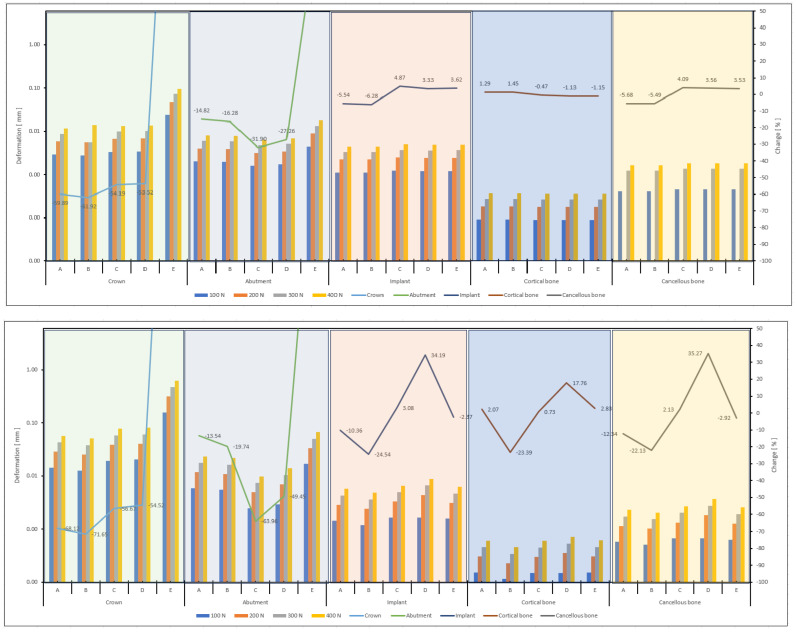
Linear graphical representations of the displacement amounts on the parts of the A, B, C, D, and E implant systems at axial and oblique forces of 100 N, 200 N, 300 N, and 400 N and the increase/decrease trends between the systems.

When the data in the graphs in Figure 8 were analyzed, the deformation trends and percentage changes of the parts and models showed significant differences. In the first graph, models A, B, C, D, and E show increasing deformation with increasing forces in the crown region. In the abutment region, the highest deformation was observed in model C and the lowest in model E. In the implant region, the deformation also increased with increasing forces, and the highest deformation was observed in model D. In the cortical bone and cancellous bone regions, the deformations were less compared to the other areas, and the lowest deformation was recorded in the cortical bone, especially in model B. In the second graph, the C model showed the highest deformation. In contrast, the B and D models exhibited a high deformation trend in the abutment region. In the implant region, the D model showed higher deformation than the other models. The B model showed the lowest deformation in the cortical and cancellous bone regions. Regarding percentage changes, the C model showed a negative trend in the cancellous bone, whereas the D model showed a positive trend.

**Figure 9:**
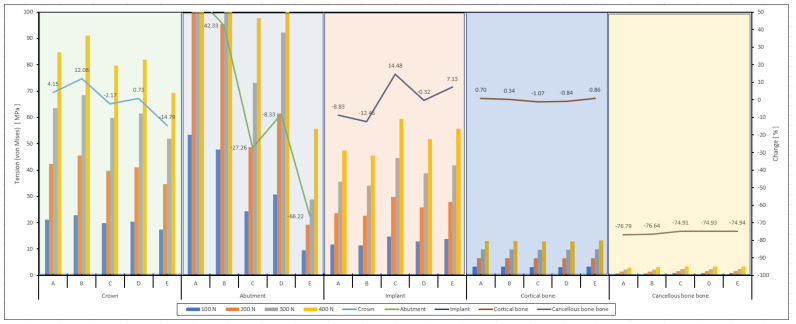

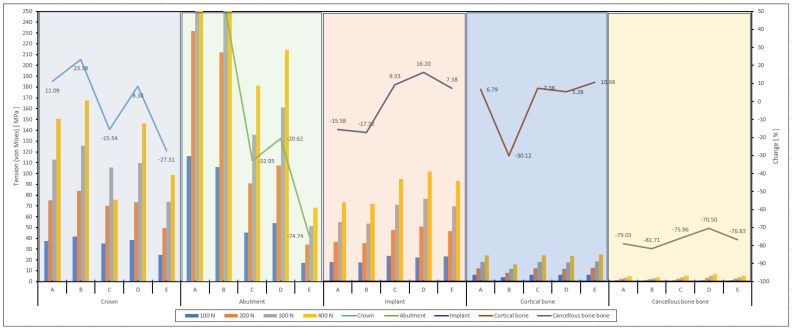
Linear graph representations of the deformation (von Mises) on the parts of the A, B, C, D, and E implant systems at axial and oblique forces of 100 N, 200 N, 300 N, and 400 N and the increase/decrease trends between the systems.

According to the graph in Figure 9, the highest deformation values were generally observed in models D and E. Models A, B, and E exhibited more deformation in the abutment region than the other models. Model D showed higher deformation in the implant region than the different models. Model E stands out regarding deformation trends in the cortical bone region. In contrast, models C and D displayed positive and negative change trends in the cancellous bone region. These results indicate that deformation trends vary across regions and models, with each model exhibiting different deformation responses in other areas.

## 4. Discussion

In this study, examining various parts and loading conditions, it was found that deformation and von Mises stress values under axial (VL) and oblique (VO) loads of 100 N, 200 N, 300 N, and 400 N showed significant differences between the abutment system models.

Stress distribution in implant systems may vary depending on the abutment selection, the abutment material, and the direction of the applied force [12,40,41]. When the analysis results of models A, B, C, D, and E (Figure 5, Figure 8 and Figure 9) were examined, it was understood that the stress distributions were concentrated on the crown, abutment, and implant. At the same time, there was less stress concentrated in the cortical bone tissue [24,42,43]. Similarly, studies in the literature have confirmed that the implant tends to concentrate in the cortical bone around the area closest to the load and the abutment neck and that there is a low-stress distribution in the cortical bone.

The data in Table 3 and Table 4 show the average deformation and von Mises stress values observed in different parts of the abutment systems under axial and oblique loads of 100 N, 200 N, 300 N, and 400 N. It is noted that model E generally had lower von Mises stress values compared to other models. Model E exhibited lower stress values in the crown (17.3 MPa axial, 34.6 MPa oblique) and abutment (9.6 MPa axial, 17.4 MPa oblique) parts, indicating less stress accumulation in these regions.

In the implant part, model E showed moderate stress levels, with 13.9 MPa under the axial load and 23.3 MPa under the oblique load, placing it at a similar or slightly higher level than other models. The stress values of model E for cortical and cancellous bone sections (3.3 MPa axial, 6.3 MPa oblique, and 0.8 MPa axial, 1.3 MPa oblique) are similar to those of the other models, showing no significant difference. Overall, model E offers lower stress values in the crown and abutment sections while demonstrating balanced performance in the implant and bone sections. This characterizes model E with stress advantages in specific regions and a balanced structure overall.

Models C and D showed the highest deformation under oblique loads at 2 µm, whereas models A and B showed the lowest values at 1 µm for both load types. Cortical and cancellous bone deformation values did not differ significantly between the models. Regarding von Mises stress, the highest stress on the crown was observed in model B (41.9 MPa oblique load).

On the abutment, models A and B had the highest stress values (116.1 MPa and 105.9 MPa, respectively). Model A showed the highest stress for the implant under axial loads (53.4 MPa), while models C and E stood out under oblique loads. Model A had the highest stress on the cortical bone, while the cancellous bone did not show significant differences between models.

When evaluating Figure 6, it is generally observed that the von Mises stress increased as the axial force increased. Models A and B had the highest stress values at each force level, while model E showed the lowest stress values with fluctuations observed at some force levels (especially 200 N and 300 N). Models C and D had lower stress values than A and B but higher than E. Different abutment designs or material properties can significantly influence stress responses. Under higher forces (300 N and 400 N), the performance of the models becomes more critical; therefore, material and design optimization should be performed to improve durability under high forces. The high-stress values of models A and B suggest that these models are more durable, but their fatigue and fracture risks should be evaluated as they can be subjected to higher stresses. According to Figure 6, the von Mises stress increased as the oblique force increased. Models A and B had the highest stress values at each force level, while model E shows the lowest. Models C and D had lower stress values than A and B but higher than E.

These findings demonstrate that the abutment design and material selection significantly impact the mechanical performance of dental implants. Similar results are observed in the literature. For instance, Tonin et al. (2023) [44] noted that the abutment material and design affect stress distribution on the implant. Additionally, Yao et al. (2019) [45] examined the mechanical performance of abutment and implant systems under different loading conditions and obtained similar results. Vinhas et al. (2020) [5] investigated the stress responses of varying abutment designs. Peng et al. (2021) [46] and Rungsiyakull et al. (2010) [47] evaluated the impact of material optimization on implant performance.

Consequently, when evaluated in terms of total or single abutment deformation values, although the displacement of the E model is relatively higher than other models since it is a viscoelastic material, it is a situation that is expected in terms of chewing suspension as we know it in natural teeth but does not exist in the current implant system. In this respect, this study has created awareness. In addition, it has been seen in the literature that elastic behavior is an expected condition in the biomechanical integrity between the implant and the jawbone [48,49,50,51].

## 5. Conclusions

According to the analysis graphs in Figure 5, the abutment (A) currently used in implant treatment had a higher stress concentration on the mounting surfaces in contact with the crown than the alternatively created abutment alternatives. Among the alternatively designed abutments, model C, with an increased surface area, will be an alternative to existing abutments in terms of homogeneous distribution of deformation and stress values (Figure 5). As seen from the graphs in Figure 5, Figure 6 and Figure 7 and Table 3, the E abutment model is in the foreground compared to all alternative models, including the current abutment, in terms of von Mises stress values at different loads. The E abutment model will be an alternative to the existing abutment model in terms of ease of assembly, application, and production.

The Kruskal–Wallis test showed significant differences between the groups (*p* < 0.001). The greatest difference was observed between models E and A (q′ = 6.215), with a significant difference also found between models C and A (q′ = 3.219, *p* < 0.005).

As a result, while the designs in this study (B, C, D, and E), which are considered alternatives to the existing (A) abutment model, create awareness in terms of applicability and ease of assembly, the abutment models C and E, which come to the fore when the von Mises tension and directions are taken into account, are the abutment models. It was concluded that it would positively contribute to the material’s life.

## 6. Patents

These designs, presented as alternatives in this respect, have no examples in the abutment models used in traditional dental implant systems that have been implemented in the past and discussed in the articles. In addition, patent application files with file number 2023/017116 have been submitted to the Turkish Patent and Trademark Office to use a viscoelastic abutment. The image below shows the patent application file dated 12 December 2023 for the viscoelastic movable abutment model.

## Figures and Tables

**Table 1:** Elasticity (E) and Poisson (ν) values were determined for the implant, abutment, and cortical bone.

Materials	Young’s Modulus (GPa)	Poisson’s Ratio
Cortical bone	13.7 [21,22]	0.3 [21,22]
Cancellous bone	1.37 [23,24]	0.3 [23,24]
Porcelain (crown)	65 [25,26]	0.25 [25,26]
Implant, abutment, screw	110 [21,27,28]	0.35 [21,27,28]
PEEK Juvora	5.5 [25,26]	0.36 [25,26]

**Table 2:** Kruskal–Wallis One-Way Analysis of Variance for abutment models (A, B, C, D, E).

Group	N	Missing	Median	25%	75%
A	42	6	11.294	6.076	21.629
B	42	6	10.079	5.420	19.303
C	42	6	5.151	2.770	9.865
D	42	6	6.492	3.491	12.433
E	42	6	2.180	1.118	4.420
Comparison		Diff of Ranks	q’	*p*	*p* < 0.050
E vs. A		2748.000	6.215	<0.001	Yes
C vs. A		1423.000	3.219	0.005	Yes
D vs. A		996.000	2.253	0.080	No
B vs. A		218.000	0.493	0.967	Not tested

**Table 3:** The sum of the average deformation values (µm) of each part of the abutment systems during axial and oblique loads of 100 N, 200 N, 300 N, and 400 N (VL: axial load, VO: oblique load).

		VL				VO			
		100 N	200 N	300 N	400 N	100 N	200 N	300 N	400 N
A Deformation (µm)	Crown	2.9	5.8	8.8	11,7	14	28	43	57
	Abutment	2	4	5.9	7.9	6	12	18	23
	Implant	1.1	2.2	3.3	4.4	1	3	4	6
	Cortical bone	0.1	0.2	0.3	0.4	0.2	0.3	0.5	0.6
	Cancellous bone	0.4	0.8	1.2	1.6	1	1	2	2
		VL				VO			
		100 N	200 N	300 N	400 N	100 N	200 N	300 N	400 N
B Deformation (µm)	Crown	2.8	5.5	5.5	11.1	13	25	38	51
	Abutment	1.9	3.9	5.8	7.8	5	11	16	22
	Implant	1.1	2.2	3.3	4.4	1	2	4	5
	Cortical bone	0.1	0.2	0.3	0.4	0.1	0.2	0.3	0.5
	Cancellous bone	0.4	0.8	1.2	1.6	1	1	2	2
		VL				VO			
		100 N	200 N	300 N	400 N	100 N	200 N	300 N	400 N
C Deformation (µm)	Crown	3.3	6.7	10	13.3	19	39	58	77
	Abutment	1.6	3.2	4.7	6.3	2	5	7	10
	Implant	1.2	2.5	3.7	4.9	2	3	5	7
	Cortical bone	0.1	0.2	0.3	0.4	0.15	0.3	0.45	0.6
	Cancellous bone	0.5	0.9	1.4	1.8	1	1	2	3
		VL				VO			
		100 N	200 N	300 N	400 N	100 N	200 N	300 N	400 N
D Deformation (µm)	Crown	3.38	6.76	10.14	13.52	20	41	61	81
	Abutment	1.69	3.38	5.07	6.76	3	7	10	14
	Implant	1.21	2.43	3.64	4.85	2	4	7	9
	Cortical bone	0.09	0.18	0.26	0.35	0.15	0.36	0.54	0.71
	Cancellous bone	0.45	0.9	1.34	1.79	1	2	3	4
		VL				VO			
		100 N	200 N	300 N	400 N	100 N	200 N	300 N	400 N
E Deformation (µm)	Crown	24	47.9	71.9	95.9	157	313	470	626
	Abutment	4.4	8.8	13.3	17.7	17	33	50	67
	Implant	1.2	2.4	3.6	4.9	2	3	5	6
	Cortical bone	0.1	0.2	0.3	0.4	0.15	0.30	0.46	0.61
	Cancellous bone	0.4	0.9	1.3	1.8	1	1	2	3

**Table 4:** The sum of the average von Mises values (MPa) in each part of the abutment systems during axial and oblique loads of 100 N, 200 N, 300 N, and 400 N (VL: axial load, VO: oblique load).

		VL				VO			
		100 N	200 N	300 N	400 N	100 N	200 N	300 N	400 N
A von Mises (MPa)	Crown	21.1	42.3	63.5	84.7	37.6	75.3	113.0	150.7
	Abutment	53.4	106.9	160.4	213.9	116.0	232.1	348.2	464.2
	Implant	11.8	23.6	35.5	47.3	18.3	36.6	54.9	73.2
	Cortical bone	3.2	6.5	9.8	13.1	6.0	12.1	18.1	24.2
	Cancellous bone	0.7	1.5	2.2	3.0	1.1	2.3	3.5	4.7
		VL				VO			
		100 N	200 N	300 N	400 N	100 N	200 N	300 N	400 N
B von Mises (MPa)	Crown	22.7	45.5	68.3	91.1	41.8	83.7	125.5	167.4
	Abutment	47.7	95.4	143.2	190.9	105.9	211.9	317.9	423.8
	Implant	11.3	22.7	34.1	45.4	17.9	35.8	53.8	71.7
	Cortical bone	3.2	6.5	9.8	13.1	3.9	7.9	11.8	15.8
	Cancellous bone	0.7	1.5	2.2	3.0	1.0	2.0	3.1	4.1
		VL				VO			
		100 N	200 N	300 N	400 N	100 N	200 N	300 N	400 N
C von Mises (MPa)	Crown	19.8	39.7	59.6	79.5	35.1	70.3	105.4	75.5
	Abutment	24.3	48.7	73.1	97.5	45.3	90.6	136.0	181.3
	Implant	14.8	29.7	44.6	59.4	23.7	47.4	71.1	94.8
	Cortical bone	3.2	6.4	9.7	12.9	6.0	12.1	18.2	24.3
	Cancellous bone	0.8	1.6	2.4	3.2	1.3	2.7	4.0	5.4
		VL				VO			
		100 N	200 N	300 N	400 N	100 N	200 N	300 N	400 N
D von Mises (MPa)	Crown	20.4	40.9	61.4	81.9	38.3	73.1	109.7	146.3
	Abutment	30.7	61.4	92.2	122.9	54.0	107.2	160.9	214.5
	Implant	12.9	25.8	38.8	51.7	22.4	51.0	76.5	102.0
	Cortical bone	3.2	6.4	9.7	12.9	6.0	11.9	17.8	23.8
	Cancellous bone	0.8	1.6	2.4	3.2	1.3	3.4	5.1	6.8
		VL				VO			
		100 N	200 N	300 N	400 N	100 N	200 N	300 N	400 N
E von Mises (MPa)	Crown	17.3	34.6	51.9	69.3	24.6	49.3	73.9	98.6
	Abutment	9.6	19.2	28.8	55.6	17.0	34.1	51.2	68.3
	Implant	13.9	27.8	41.7	55.6	23.3	46.6	69.9	93.2
	Cortical bone	3.2	6.5	9.8	13.1	6.2	12.5	18.8	25.1
	Cancellous bone	0.8	1.6	2.4	3.2	1.3	2.6	3.9	5.2

## Data Availability

Data will be made available on request.
